# SeniorHealth Tracker application to support the elderly: technological innovation leveraging humanisation

**DOI:** 10.3389/fdgth.2026.1743131

**Published:** 2026-02-27

**Authors:** Juliana Basulo-Ribeiro, Ana Ferreira, Leonor Teixeira

**Affiliations:** 1 Department of Economics, Management, Industrial Engineering and Tourism (DEGEIT), Institute of Electronics and Informatics Engineering of Aveiro (IEETA), Intelligent Systems Associate Laboratory (LASI), University of Aveiro, Aveiro, Portugal; 2 RISE-Health, Department of Community Medicine, Information and Health Decision Sciences, Faculty of Medicine, University of Porto, Porto, Portugal

**Keywords:** elderly empowerment, health technology, mHealth, people empowerment, social determinants of health, user-centred design

## Abstract

The advance of medicine and technology has been a catalyst for the population's longevity, reflected in the increase in elderly citizens. However, this increase also comes a burden on caregivers. To address this gap between need and accessibility, the motivation for this study arises, highlighting the challenges faced by an ageing population. This work presents a preliminary proof of concept of an innovative digital tool (a mobile app prototype) to support older people to live more independently and safely, while facilitating communication between them, caregivers and health professionals. To develop the prototype for monitoring elderly health, the User-Centred Design methodology was applied, concluding with a usability evaluation. As a proof-of-concept, this study suggests that combining technology with human support may contribute to improved elderly care and empowerment; however, these implications remain preliminary and require validation in larger and more diverse evaluations. Theoretically, it uses a social determinant of health lens to outline potential ways in which health apps could support access to care in this age group, to be examined in future, larger-scale evaluations. From a practical perspective, it contributes with a preliminary proof of concept and prototype offering use-cases aimed at improving the quality of life of this population.

## 1 Introduction

### 1.1 Contextual background

In the fourth industrial revolution, we are witnessing an unprecedent transformation driven by technology, with no sector remaining untouched, and health is one of the sectors where digitalisation could have the greatest impact. Technological advances promise an era of innovations capable of expanding the frontiers of human life, democratize access to care, and personalize medicine like never before (1, 2). In this scenario of revolution, technology is not just a tool, but a vital ally in improving quality of life, health and well-being.

During the COVID-19 pandemic, there has been a significant increase in digital health, including the use of mobile health apps, telemedicine and data analysis to improve healthcare systems (3). As such, it is important to recognise that mHealth has the potential to empower individuals by improving personal health management, offering more immediate and personalised support, which not only improves adherence to treatments but also fosters patients' active involvement when it comes to their long-term well-being (3, 4). In addition, it can provide services in underserved areas (5, 6). However, this technology also places greater responsibility on the individual for the prudent use of health resources. There has been an increase in the use of smart devices for health monitoring, supported by substantial investment, although there are concerns about data privacy. Thus, the growing demand for reliable personal health apps is clear, with the potential to expand as technology continues to evolve (7, 8).

As the use and availability of mobile health apps increases, so does the need for a comprehensive and accessible app evaluation framework (9). Therefore, the search for a suitable and safe mobile health app presents a challenge, as the market offers hundreds of solutions, making it difficult for the user to filter the choice of those that are validated by health professionals (4). In this context, Tabi et al. (4) concluded in their study that of the 328 apps categorised, the majority were developed by the software industry (73%, 11/15), while a minority were co-developed with healthcare professionals (15%, 3/20) or academia (2.1%, 7/328). These data emphasise the importance of involving health professionals in the development of apps to ensure their clinical relevance and validity.

Results from a recent survey indicate that approximately 40% of adults use health apps and 35% use wearable devices, with the majority using these technologies daily (10). This raises concerns about how these data are stored, interpreted, and integrated into clinical decision-making. In fact, although many people already use wearable devices and apps to monitor their health, there is still uncertainty about how this data can be effectively analysed by qualified professionals and converted into useful information for patient care. This highlights the need for interdisciplinary collaboration between doctors, patients and developers to maximise the potential of these technologies (11, 12). Through interviews with 11 healthcare professionals in a previous study (13), it was found that they are aware that it is essential to develop technologies that meet the needs of clinical practice from the outset, led by specialists and with the participation of all stakeholders, to promote interdisciplinary collaboration.

In line with the broader Industry 5.0 vision for healthcare, the literature emphasises that the adoption and meaningful impact of emerging digital solutions depend strongly on user acceptance and inclusive, human-centred design (14). This reinforces the need to understand end users' characteristics and contexts when developing mHealth and wearable-based interventions. The continuous advance of medicine and technology has proved to be a catalyst for population longevity, a global trend that is reflected in the annual increase in the number of elderly citizens in most countries. While technology has provided advances in human life and medical care that have extended life expectancy, it has also intensified other challenges (15).

Between the advances promoted by technology and the increase in life expectancy, a vacuum emerges in which social and medical challenges intensify. This gap is especially visible in the tension between the capacity of health services and the growing demand for care due to an ageing population. In the face of this gap, technology is emerging as an essential link, not only for extending years of life, but also for promoting quality of life. Digital innovations are increasingly being seen as an indispensable resource for providing scalable and effective solutions that can act as a link between healthcare professionals, informal caregivers and patients, improve data communication and promote a process of mutual and continuous learning, contributing to the promotion of patient health and the prevention of risks (diseases) (14, 16).

Therefore, with the demand for care services increasing every year and limited resources to support them, the elderly population urgently needs solutions that promote their autonomy in everyday life (17). In line with the above, studies on the application of technology in the health of the elderly have revealed that apps can help healthcare professionals remotely monitor the health status of older people and improve the self-management of elderly patients in the comfort of their own homes (15, 18).

Health 4.0 promotes a health service delivery model that is coherent with the fundamental principles of Society 5.0, emphasising a vision for a new societal model to achieve a human-centred society (16). In this scenario, when considering the care landscape for the elderly population, the social determinants of health (SDH) take centre stage, highlighting the need to go beyond mere medical care (19, 20). These determinants, which encompass economic, social, cultural and environmental factors, have a decisive influence on living conditions and, consequently, the health of older people (21). Therefore, when developing technological solutions, it is essential to incorporate an understanding of these factors to promote not only autonomy but also equity in access to health. The strategic use of technology within the context of social determinants has the potential to mitigate disparities, contributing to healthy ageing and a more inclusive and just society (22).

### 1.2 Study motivation & goals

A previous study based on interviews with 11 health professionals, offered a twofold possibility regarding the influence of technology on the elderly population:

On the one hand, it can lead to greater isolation and contribute to a disparity in access to technological innovations and their benefits, which is also mentioned in the literature (23). However, other authors point out that technology can prevent social isolation (24).
On the other hand, the professionals interviewed assume that technology can significantly improve the accessibility and quality of healthcare, thus contributing to the promotion of equity in access to healthcare services, as has been discussed in the literature (25–28). In this sense, the literature points to a positive contribution of technology to the Social Determinants of Health (20).

In addition, the study by Bhayana et al. (29) revealed that there is a technological gap between the older and younger generations, and, for this reason, comprehensive and contextualised solutions are needed to address the well-being of the elderly. Furthermore, the study by Kim et al. (17) highlights that in 2020 there were around 727 million people aged 65 and over, a figure that is expected to reach 1.5 billion by 2050, increasing the need for digital solutions that promote not only autonomy in the daily lives of the elderly, but also the need for care services, highlighting also the importance of technology to support family members and caregivers to ease their burden.

This study is motivated by the significant challenges presented by the growing ageing population, which affect both older individuals and their caregivers (this motivation derives from both previous literature and previous studies carried out by the authors). Given the gap between existing needs and the accessibility of solutions, it is crucial to develop technologies that respond to these specific challenges. Therefore, this study aims to create and present a preliminary proof of concept of an innovative digital tool (prototype of a mobile app) to support older people to live more independently and safely, promoting self-care and personal empowerment, while facilitating communication and coordination between the older person, caregivers and health professionals. This solution represents an innovative digital tool for monitoring the elderly, designed with the involvement of a group of users (the elderly), their caregivers and some health professionals. In addition, through these functionalities were designed considering SDH as a rationale and may potentially support inclusion and equitable access to healthcare, as well as reducing isolation.

Finally, a targeted literature review was conducted to frame the problem space and inform the initial design directions of the prototype; the key themes identified are synthesised in Section 2 (Theoretical Background).

## 2 Theoretical background

### 2.1 Humanised digital health for ageing populations

The integration of digital health technologies into the care of older adults has considerable potential to enhance quality of life, reduce social isolation, and improve the efficiency of healthcare delivery. Yet these benefits largely depend on how such technologies are designed and implemented (30). Humanisation in digital health seeks to ensure that technology does not reduce patients to data points but instead acknowledges and respects individual needs and lived experiences (31). While this concern applies across contexts, it is particularly relevant in ageing populations, as many older adults live with multimorbidity, increased vulnerability and, in some cases, social isolation—factors that may heighten anxiety when engaging with digital tools (30, 32). In this sense, digital health should complement care rather than replace it, supporting communication, decision-making and timely responses (33).

To translate this humanised vision into practice, User-Centred Design (UCD) is essential (34). UCD places end users at the core of development by involving them from the early stages to understand their needs, tasks and contexts of use. It relies on iterative cycles of prototyping and usability testing, allowing solutions to be refined progressively through feedback and observation of real-world use (35–37). Practically, this may involve simplifying and clarifying language, reducing the number of steps, optimising icons and visual elements, and improving readability through appropriate font size and contrast. UCD also helps address a recurring limitation in research and technological development: the tendency to treat older adults as a homogeneous group, often automatically associated with cognitive decline, frailty and dependency. Ageing, however, is highly heterogeneous, and overlooking this diversity can result in poorly fitted and less inclusive solutions (30). In older populations, this is closely linked to accessibility: simple interfaces, legible text, good contrast, clear feedback and low cognitive load can increase trust and sustained use (38–40). Accordingly, Gomez-Hernandez et al. (38) propose two “golden rules” for designing mobile applications for older users: simplify, and increase the size and spacing of interactive controls.

Even so, anxiety related to digital technology use is common among older adults and may be shaped by low digital literacy, fear of damaging devices, and previous negative experiences (41, 42). Therefore, inclusive design is not only a usability matter: it can also reduce digital literacy barriers and help prevent innovation from widening inequalities in access to healthcare, namely the digital divide (43–45).

Finally, aligning humanisation, ageing, UCD and digital health requires careful attention to trust, privacy and data sharing, since many real-world solutions involve multiple users with different needs and levels of access (46). A humanised approach should ensure transparency (what data are collected and why), control (who can access what), and simple, clear rules for risk alerts, balancing safety with autonomy (47–49). This balance is particularly critical in ageing populations and, when embedded from the outset of design and implementation, tends to make technologies more acceptable, safer, and better aligned with person-centred care, supporting higher-quality ageing (50).

### 2.2 mHealth apps for elderly people

As mentioned above, mobile health apps are emerging as powerful tools to support self-care, particularly relevant for the elderly population. These applications offer a diverse range of functionalities and stand out for their ability to: allow patients to self-manage their health; provide users with relevant information about their state of health; promote real-time communication between patients and health professionals; enable a rapid response to significant changes in the patient's health condition, among others (51, 52). To help design technological solutions that are passive and accepted by the elderly, Wilson et al. (53) highlight in their study the barriers and facilitators to the use of technologies by the elderly population.

Regarding the health of the elderly, the literature proposes various apps designed to monitor and improve various aspects of the health and well-being of this age group in a specific way. Table 1 shows some of these apps.

**Table 1:** Use cases found in the literature.

Use-Cases	Description	References
Fitness and Exercise	Application that facilitates physical exercise adapted to people with limitations.	(54)
Prevention and Detection of Falls	Apps dedicated to the prevention and detection of falls, essential for the safety of the elderly in their daily lives.	(55, 56)
Well-Being	App that aims to improve health and mental well-being of the elderly.	(57–59)
	App that promotes mindfulness practices adapted to the needs of the elderly, helping to reduce stress and improve well-being.	(60)
	App that improves the social and physical well-being of the elderly, facilitating their independence and connectivity with loved ones, healthcare professionals and the community.	(29)
Adherence to medication	App that helps the elderly to manage their medication, ensuring adherence to the prescribed treatment.	(61, 62)
Nutrition	App that provides personalised nutritional guidance, promoting a healthy diet suited to the needs of the elderly.	(63, 64)
Vital Signs Monitoring	App that allows continuous monitoring of vital signs, crucial for early detection of potential health problems.	(29, 65)
Applications for Nursing Homes and Day Care Centres	App that offers solutions adapted to the nursing home and day centre environments, improving the management of the health and well-being of the elderly in this setting.	(62)
Monitoring Specific and Chronic Diseases	Apps developed to monitor and manage specific or chronic illnesses, personalising care and improving the quality of life of the elderly.	(62, 66)

### 2.3 mHealth impact on SDH (social determinant health)

The impact of mHealth on SDH is significant and multifaceted, with the potential to transform and improve public health practices (19, 20). The literature identifies different areas in which SDH can be impacted by mHealth, emphasising the following:

**Improving access to healthcare:** mHealth can improve access to healthcare services, especially in remote or underserved areas, by enabling remote consultations, health monitoring and delivery of health information. This can help reduce geographical and economic barriers and motor disabilities, allowing for more equitable access to healthcare (19, 20, 54, 67, 68).
**Mitigating socio-economic inequalities:** mHealth interventions can offer less-costly, large-scale solutions, making them ideal for improving health parameters in developing countries by promoting health equity. This has significant implications for DHS, as it could help alleviate poverty-related diseases and improve equity in access to health services (20).
**Patient education and empowerment:** mHealth can empower patients by providing accessible and understandable health information, encouraging self-management of their health (67, 68).
**Disease monitoring and prevention:** mHealth applications that enable continuous health monitoring can help with the prevention and early management of medical conditions, facilitating personalised and timely interventions that can respond to individual health needs (54).
**Collecting and analysing health data:** mHealth offers a platform for collecting health data in real time, which can improve public health surveillance and evidence-based decision-making. This can help identify and address the social determinants of health at community and population levels (19, 69). Willingham et al. (54) discusses the concept of precision care as a future direction, in which treatment strategies are tailored to an individual's specific needs, abilities and goals.

Despite these benefits, some authors also identify challenges. Hendl & Shukla (68) emphasise that while digital health technologies offer the potential for widespread access and the empowerment of patients to monitor their health, they also entail existing inequalities and sometimes do not produce equitable health outcomes.

In addition, Paglialonga et al. (5) introduce the concept of the “digital divide”, which highlights disparities in access to and use of digital technologies, such as mobile health (mHealth) apps and devices. These disparities can be influenced by various factors, including, age, location, socioeconomic status, education and digital literacy. The concept therefore emphasises the importance of measures to ensure that technologies are accessible to all, to reduce health inequalities and ensure that advances in healthcare benefit the entire population equally.

## 3 Methods

To achieve the goal of developing a prototype of a digital tool to support the health monitoring of elderly people, the User-Centred Design methodology was applied, as this increases the likelihood of user adoption and satisfaction with the technology developed (70).

The study involved three stakeholder groups: (i) older adults (end users), (ii) caregivers, including informal caregivers (family members) and formal non-clinical caregivers (e.g., nursing-home or home-care staff), and (iii) registered nurses (general nurses and nurses specialising in geriatrics), who contributed as expert stakeholders during the refinement phase. In total, 13 different participants were involved: 4 older adults, 4 caregivers, and 5 registered nurses (including 4 interviewed as a separate expert group and 1 participating in the usability sessions).

Initially, we conducted a targeted (narrative) literature review to frame challenges related to demographic ageing and older adults' health monitoring, and to identify design considerations relevant to caregiver-supported mHealth solutions. The review was intended to inform early-stage prototyping (rather than to provide a systematic synthesis of evidence) and guided the subsequent user research and initial requirements definition.

### 3.1 Requirements survey

The literature review was followed by the user research stage, where the needs and requirements of both parties (elderly people and their caregivers, as the application has 2 different users) concerning the app were collected and understood so that it could be better aligned with the demands and expectations of the end users. As Gomathi & Mishra (14) point out, the success of digital health technologies depends largely on user acceptance. It is crucial to understand the profile of users and professionals to integrate technology inclusively. To carry out the user research, questionnaires were initially carried out for the elderly and their caregivers. However, it was concluded that this would not be the best instrument, as the language might not be easy to interpret for all elderly people and caregivers, even if it were adapted. The decision was therefore made to conduct informal interviews with both parties involved in the app. These interviews, with both parties (senior + caregivers), followed two different scripts, each based on the same three fundamental axes: initially, demographic information was collected to contextualise the responses within the variables of age, location and preference for health institutions. Next, the preferences and usage habits of health apps were analysed, seeking to understand which functionalities were most appreciated and how regularly they were used. Finally, the identification of specific functionalities desired by the users was explored in depth, including their relevance and suitability to their daily needs, with the aim of optimising health and well-being support through digital technology.

Prior to the interviews, participants received information about the study aims and how the data would be used. Written informed consent was obtained, and participation was voluntary. Participants were recruited through convenience sampling based on accessibility and willingness to take part and included older adults and caregivers with relevant experience. Interviews were informal semi-structured, and data were captured through researcher field notes (no audio-recordings). Confidentiality and anonymity were ensured; no directly identifiable personal data were collected, and notes were anonymised prior to analysis in line with GDPR requirements. The same information and consent procedures were applied to the usability testing sessions, with informed consent obtained prior to testing and no identifiable data recorded. Ethical review and approval were not required for this study in accordance with local legislation and institutional requirements.

The analysis was conducted by the first author using an inductive thematic synthesis approach appropriate for formative UCD studies. Interview data were synthesised to develop personas and requirements that guided the design and development of the application. Field notes were reviewed iteratively across participants to identify recurring needs, barriers, preferences, and desired functionalities, which were clustered into broader themes (e.g., medication adherence support, ease of navigation, reassurance/monitoring, and caregiver coordination). Personas (older adult and caregiver; Section 4.1.1.1) were derived by consolidating recurring participant characteristics (goals, motivations, frustrations, context of use, and digital confidence). Themes were translated into functional and non-functional requirements (Table 2) and mapped to the user scenarios/use cases (Section 4.1.1.2), supporting the definition of the app's functionalities and attributes. Given the small, early-phase sample and single-researcher synthesis, the personas and requirements should be interpreted as preliminary design inputs to guide prototyping rather than as exhaustive or generalisable user profiles.

**Table 2:** User research results.

Category	Elderly responses	Caregiver responses
Use of health apps	25% of the elderly interviewed use health apps, but there is a general openness to using them	50% use health apps to monitor themselves, 50% are interested in using them, focusing only on validation of the apps by health professionals.
		To organise information about the elderly, some caregivers use notes written on their mobile phones or methods written on paper, but all are open to using specialised apps for this purpose.
Technology acceptance	General openness to the use of technology, including apps and wearable devices.	Open to using specialised apps for care management.
Frequently used applications	Everyone uses apps, and the most used are Whatsapp (75%) and Facebook (50%).	N/A
Health data to be monitored	Blood pressure (100%), heart rate (75%), blood oxygen (50%), temperature (25%).	Blood pressure, blood oxygen, temperature, heart rate.
	Everyone is willing to share this data with the caregivers.	
Cause for not taking medication	It was realised that forgetfulness and not having the medication with them at the right time were common factors that prevented all the interviewees from taking their medication, highlighting the importance of reminders in the app and a way of carrying their medication with them. (Some of the elderly even said: “I'm in the countryside and I remember to check the time” or “if I've left the house and I don't have them with me, of course I'm not going to take them”).	They emphasise the importance of personalised reminders to prevent medication failure, suggesting forgetfulness as a cause for not taking medication.
Medication management	General need for reminders; different preferences for the type of alarms (visual, sound and vibration), suggesting that the app should have customisable options	Medication management as the main feature, personalisation of the medication plan from the doctor, medication history for efficient and accurate monitoring.
Pharmacy assistant	They value home delivery of medication. They say it saves them from having to “bother” family members or walk alone.	They recognise the importance of a pharmacy assistant who receives the prescription directly from the hospital and contacts the elderly person to deliver the medication to their home.
Wearables	50% wear smart watches, 50% are willing to wear them. Devices must be practical and discreet.	N/A
Virtual assistant	Valued for providing company.	Interest in a reliable chatbot to answer questions about healthcare for the elderly. 75% considered it important to do so via the app, and 25% spoke of their preference for human contact.
Design preference	Bright colours, simple and clear design	High-contrast colours for easy reading.
Accessible local services	Hospitals, health centres, pharmacies (100%); physiotherapy centres (25%), supermarkets (50%). (Some of the expressions used to reinforce this need: “the family can't go everywhere with us…they have their own lives, and we have to get around as best we can.”)	N/A
Emergency button	Useful for emergency situations when you're alone.	Crucial, especially for elderly people who live alone and are autonomous; the need for a virtual assistant to verify the need for help, which asks the elderly person if they really want to ask for help, or if it was a mistake, so that the alert becomes more reliable.
Integration with services	N/A	Desired integration with local emergency services for fast and efficient response.
Information and education	Interest in tips on healthy living in old age (adjusted to the objective set by your doctor) and how to act in abnormal health situations.	Similar to the elderly, with a focus on healthy living tips and managing health conditions.
Alerts and notifications	Medication reminder	The importance of receiving customisable alerts, depending on the needs of each caregiver, for each elderly person. All the caregivers interviewed (100%) valued receiving alerts when the monitored values were outside normal thresholds or when there were failures to take medication. 75% of caregivers consider it crucial to receive notifications in emergencies triggered by the elderly person pressing the emergency button. 25% of the interviewees emphasised the importance of being alerted in the event of a fall.
Localisation tracking	N/A	Real-time location of elderly people, history of places visited by 75% of caregivers.
Functionalities considered most useful	Medication reminders, Alert button, Management of vital signs (simplified), Virtual assistant.	Medication management, Need for support with queries, Information on the elderly person's location, Emergency alerts, Monitoring vital signs.

N/A, not applicable.

Among the elderly participants interviewed (*n* = 4), participants were aged between 72 and 78, with an equal geographical distribution between the Centre and North regions of Portugal. Regarding access to healthcare, there is a tendency to use public services, with half of the interviewees using them exclusively. Among the caregivers interviewed (*n* = 4), participants were aged between 47 and 60 years, and the group includes caregivers from the Centre and North regions of Portugal. Their experience in caring for the elderly ranges from 7 to 12 years, demonstrating considerable commitment to their profession.

This phase was conducted as an early-stage exploratory User-Centred Design activity. Therefore, a small convenience sample was intentionally used to obtain initial qualitative insights and inform personas, scenarios, and the first prototype iteration, rather than to achieve demographic representativeness.

### 3.2 Prototype development

The subsequent phase focused on drawing up wireframes and workflows, graphic representations of the path to show the user's interaction flow within the application. An agile, iterative and incremental methodology was adopted at this stage of the model's development, allowing it to be implemented while users evaluate the tool and add requirements. This prototype is a mockup created using Figma design software.

Accessibility was treated as an essential design requirement related to the age of future users, which may affect interaction with mobile technologies (e.g., reduced visual acuity, cognitive load, motor skills, and digital literacy). Based on the information gathered about the requirements, the interface prioritised a simple and clear layout, with large font, high-contrast visual elements and the use of colour as a supporting cue rather than the sole carrier of meaning. In addition, multimodal interaction was considered important: data informed the inclusion of voice-based interaction and different alert modalities (visual, audible, and vibratory) to support medication routines and emergency situations. Finally, a voice-operated virtual assistant was incorporated to align with the usability, support, and companionship needs expressed during the requirements-gathering phase. These choices were informed by interview findings and direct observation, which guided the selection of colour and voice-based interaction elements for the prototype.

### 3.3 Testing the prototype

After the first cycle, we carried out a preliminary evaluation that revealed key areas to adjust in order to improve the usability and acceptance of the app. Feedback for these cycles came from three caregivers participating in the evaluation, who provided valuable insights into the interface and functionality. After implementing the improvements suggested in the first cycle, a second cycle was required for further refinement and to ensure that all concerns were adequately addressed. The same caregiver participants from the interview phase participated in both prototype-testing cycles, ensuring continuity of feedback and enabling verification that the refinements implemented after Cycle 1 addressed the issues identified.

Following these two iterative cycles and the finalisation of the prototype, usability tests were carried out individually, with the aim of assessing the application's acceptance by the two types of users, validating all the requirements defined in the user research and measuring the application's usefulness. Users were asked to complete a series of specific tasks, while being observed as they carried them out, to analyse the ease of use and efficiency of the application.

Tasks were selected to reflect key and safety-relevant use-cases identified during the requirements-gathering phase (e.g., medication confirmation, checking health status and contacting the caregiver, setting a medication routine, and reviewing adherence history). Sessions were conducted in participants' homes, lasted approximately 30 min, and used an interactive, moderated task-based procedure on a smartphone prototype. Participants interacted with the prototype while the moderator observed performance and asked brief clarification questions when needed. At the end of the session, participants completed a short *ad hoc* post-task usability questionnaire developed for this formative evaluation (i.e., not a standard instrument such as SUS). The questionnaire used Likert-style ratings with an “NA” option and included two sets of items: (1) overall opinion about the application (e.g., ease of orientation, findability, perceived slowness, pleasantness, coherence/consistency, and need for help); and (2) interface-specific aspects (e.g., readability/font size, emphasis of important information, adequacy of information layout across screens, icon intuitiveness, and ease of navigation). Open-ended questions were also included for additional comments.

The first author acted as moderator/observer, providing standardised task instructions and recording difficulties during task completion (e.g., hesitations, navigation errors, misunderstandings, and requests for help). Below are some examples of tasks performed, focused on functionalities for the elderly:

Imagine that you need to check that your health figures are in order, to understand whether you need to call the caregiver or not. Is everything OK or do you need to call the caregivers?
Confirm that you have taken your medication when you receive the notification in the app.

For healthcare caregivers, the tasks included:

Add the medication routine for “paracetamol” with the following characteristics:
  • medication: “Paracetamol”;
  • dosage: “2” + “tablets”;
  • route of administration: “oral”;
  • when: “Continuous use”;
  • from: “05 June 2024”; until: “05 July 2024”;
  • - time: “10h” and “18h”.
Review the older adult's medication history and check whether all medication was taken correctly on 03 July 2024.

Each task was assessed by users for its ease of completion using a Likert scale, where 1 represents “not easy at all” and 5 stands for “very easy”. At the end of the tasks, both elderly and caregivers were asked to answer a questionnaire to give their opinion of the application's usability. Scores were analysed descriptively to identify usability friction points and prioritise interface refinements, with lower ratings indicating tasks requiring redesign or clearer guidance.

A total of 13 usability test sessions were conducted. Of these, eight focused on the older-adult user flow: three were performed by older adults (70–80 years), and five were performed by caregivers to assess the usability of the older-adult interface. The remaining five sessions focused on the caregiver flow and were performed by five participants aged 40–60, comprising the four caregivers previously interviewed plus one registered nurse, who also tested the caregiver interface.

The elderly completed 6 tasks, the aim being to ensure that the interface was intuitive and easy to use, considering possible technological or cognitive limitations common in this age group. On the other hand, the caregivers completed 7 tasks related to the part of the application aimed at them, and these tasks were designed to assess how the application can help in their caring responsibilities, ensuring that the functionalities offered are practical and efficient for supporting the elderly. In addition, as mentioned above, the caregivers also carried out the 6 tasks for the elderly. This was done to capture a support perspective and anticipate how caregivers might assist older adults during adoption and daily use.

Between August and September 2024, we additionally conducted four informal online interviews with a separate group of registered nurses (generalist and geriatric-specialist), mainly from the Aveiro and Porto regions. These participants did not take part in the caregiver/user usability sessions. Their feedback informed refinements to both the prototype features and the underlying data structure (e.g., how medication- and health-related information were organised and represented), supporting validation of the proposed approach. In addition, these expert inputs helped shape the future work directions outlined in the manuscript, particularly regarding clinical validation and further refinement of data representation.

Based on the feedback gathered from all of these participants, final adjustments were made, culminating in the final version of the SeniorHealth Tracker app prototype presented in Section 4.1.2.

**Figure 1:**
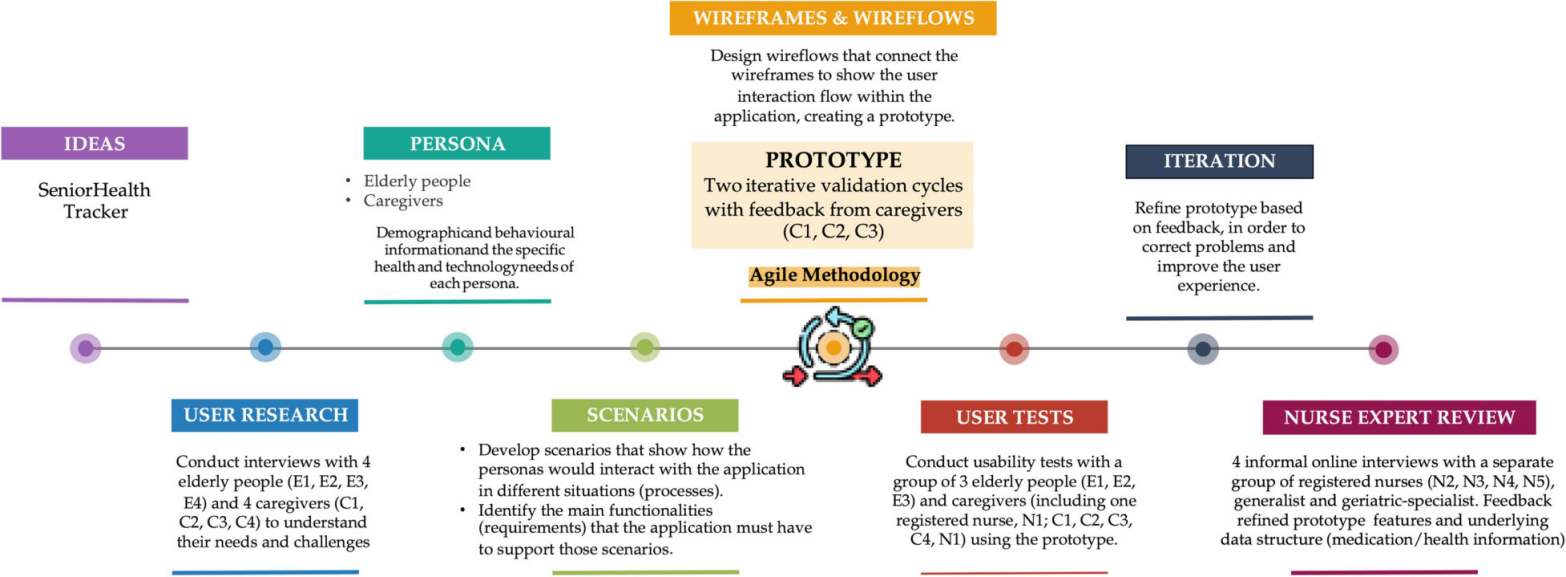
Methodology to achieve the objective.

Figure 1 summarizes the steps of the methodology used in this study.

## 4 Results

This section is divided into two parts. The first part (4.1.) presents the results of the SeniorHealth Tracker app prototype development process. The second part (4.2.) explores potential pathways through which the app prototype may influence the social determinants of health.

### 4.1 mHealth—SeniorHealth Tracker

This subsection is divided into two specific parts. The first (4.1.1.) presents the requirements for developing the solution, which came from the user research. The second (4.1.2.) presents the final version of the app prototype with the adjustments made according to the comments from the usability tests.

#### 4.1.1 Description of solution requirements

The description of the solution's requirements, based on the interviews conducted with the elderly and their caregivers, is specified in this section. Firstly, the responses from both groups of interviewees are detailed, which resulted in the app's personas. Finally, the functionalities included in the app will be detailed.

By analysing the answers provided by the elderly and caregivers interviewed, we can draw various conclusions about their preferences and needs for a health app. Table 2 presents the main results, for each category comparing the perspectives of the elderly and caregivers.

**Figure 2:**
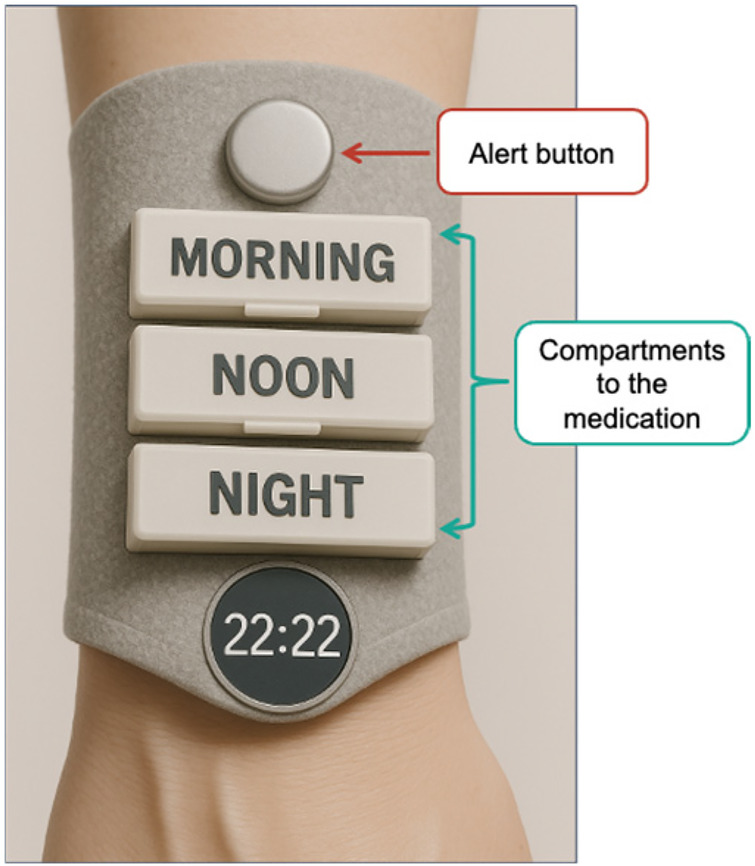
Wearable device prototype—wrist pouch.

In addition to the interviews, and after they had been completed, the authors carried out direct observation of two elderly people using apps. It emerged that colours (the visual part) and voice are the best ways to attract them. In addition, the interviews revealed the need to adapt the design of the wearable: initially conceived as a waistcoat, the participants expressed concerns about comfort, saying that it would be uncomfortable. In response to this feedback, the device evolved into the shape of a bracelet to be worn on the wrist. This new configuration, similar to a more elongated watch, was designed to accommodate both an emergency button and dedicated compartments for medication, aligning practicality with a more subtle and comfortable design (see Figure 2).

Also, the elderly expressed strong interest in a voice-operated virtual assistant. While this is a preliminary signal from a small exploratory sample, it may reflect openness to digitally mediated support among some older adults.

##### 4.1.1.1 Personas

The user research resulted in the personas of the elderly person and the caregiver, explained below.

As for the **elderly persona**, Narciso is an autonomous 75-year-old who appreciates his independence and keeps active by walking and gardening. He integrates technology into his daily life to stay in touch with family and friends. His motivations include personal autonomy, safety and the convenience of using technology adapted to his needs. Narciso's goals are to maintain a healthy and independent lifestyle, monitor his health easily and take his medication strictly on time. However, he faces frustrations due to his advancing age, which leaves him dependent and insecure, as well as the difficulty he feels in dealing with technologies that are not intuitive, resulting in a loss of social contact.

As for the **caregiver persona**, José Almeida is a 52-year-old health caregiver, a professional with experience in leading teams and managing complex projects. About 10 years ago, due to a family situation, he decided to apply his organisational skills to caring for the elderly, focusing on their well-being. He uses little technology to keep up to date with the state of health of the people he cares for and looks for innovative solutions to improve their quality of life. He wants to offer personalised support and monitor the health of the elderly efficiently, as well as control and manage their medication more effectively. However, he is frustrated by some inefficiencies in the process of monitoring the elderly, delays in responding to emergencies due to a lack of up-to-date information and a shortage of technological resources adapted to the elderly.

##### 4.1.1.2 Use-cases

Table 3 shows the features and/or interaction scenarios to be incorporated into the app, designed for both groups: the elderly and caregivers. Each group will have access to six features for each of them, selected to meet the needs addressed during the interviews.

**Table 3:** SeniorHealth Tracker functionalities.

Functionality	Elderly description	Caregiver description
Health Monitoring	You want to measure the vital signs: blood oxygen level; heart rate; temperature; and blood pressure. A green or yellow circle should appear on the screen. If it's green, everything is fine, and if it's yellow you should call the caregiver.	You receive immediate alerts for any abnormal readings, allowing you to respond quickly to potential medical emergencies. You can consult the health values of the elderly people you care for in real time.
Medication Management	A reminder should appear on the screen when it is time to take the medication, and the compartment in the wearable, illuminated with blue light, opens and the elderly person must take the medication and confirm this action on the mobile phone. If they don't confirm, the mobile phone waits 5 min, and at the end of that time, the virtual assistant asks if they have taken their medication, reminding them.	Allow the caregiver to have real-time access to information about the elderly person's medication. They can establish and edit the elderly person's medication routine based on the prescription information provided by the hospital, which communicates directly with the app. In addition, the caregiver can also choose the assistant's pharmacy.
	In addition, the elderly person can fill in the assistant pharmacy, a pharmacy that will be notified once the prescription has been made. The pharmacy will then call the elderly person and ask if they would like to receive the medication at home.	
Virtual Assistant (Chatbot)	This virtual assistant offers companionship, reducing isolation and helping to navigate the app. It can initiate conversations if it notices that the elderly person is alone and awake, and can connect the elderly person with other people or services if necessary.	A virtual assistant based on the GPT model, which answers technical health questions following care guidelines for the elderly. It allows you to offer continuous support, clarifying doubts about medication administration, common symptoms, preventive care, among other health issues.
Local Services Mapping	Inclusion of a directory of local services, such as health centres, pharmacies and hospitals, with the possibility of requesting transport. After requesting transport, a virtual assistant should start a conversation, saying: “5 min until the driver arrives, prepare your mobile phone, identification card, and others” and keep talking until the driver arrives.	N/A
GPS Location	N/A	It allows caregivers to monitor the elderly person's location in real time and access their location history. This is essential for ensuring the safety of the elderly, enabling a rapid response in the event of an emergency and giving caregivers peace of mind by being able to locate the elderly person at any time.
Health and Well-being Tips	It allows you to consult a care plan with personalised tips, according to your objectives, and tips on how to live healthily in old age, as well as instructions on how to proceed in the event of abnormal health values.	
Alert Button	Alert button to help keep the elderly safe. When pressed by the elderly person, the button notifies caregivers to ensure a rapid response.	The caregiver receives a notification with the elderly person's location and health figures. When the notification is received, the caregiver makes a phone call to the elderly person. If the first contact doesn't answer, the caregiver calls the elderly person's home. If the first two attempts fail, a close relative is notified with a phone call.
		If the caregiver finds that the health figures are well outside the normal range, the caregiver can notify the emergency services directly, guaranteeing fast and effective assistance. If this is not the case, the caregiver goes to the location mentioned in the notification.

N/A, not applicable.

#### 4.1.2 Final version of the SeniorHealth Tracker app

The tool can be used by 2 types of users: the elderly person and the caregiver. Each of them has different functions in the application. Some of the application's screens and functionalities are shown below, along with an explanation of what each one represents. The following interfaces are the result of the development carried out and have been validated through usability tests, already incorporating the suggestions for improvement made by the participants.

##### 4.1.2.1 Elderly

**Figure 3:**
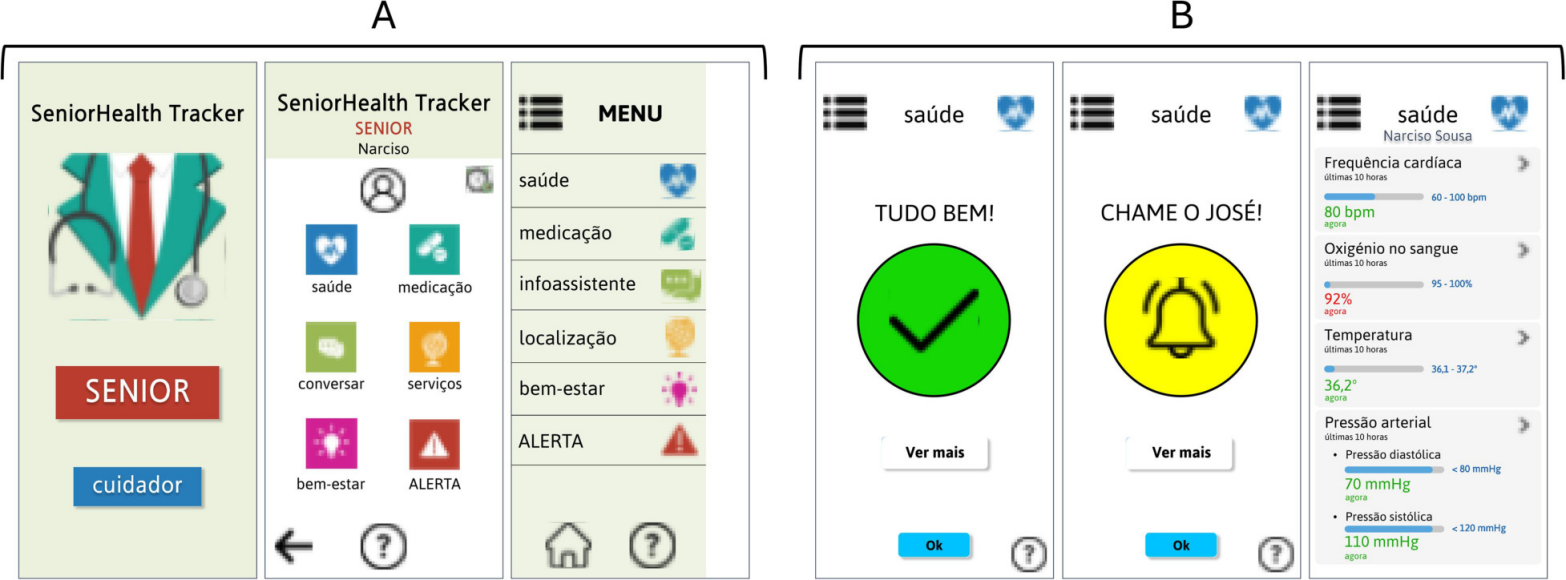
**(A)** Elderly initial menu and lateral bar; **(B)** Health monitoring module. (Interfaces in Portuguese).

Figure 3A shows the initial screen, which is the main entrance to the application, where the elderly person chooses SENIOR and then goes to the menu with the different functionalities to choose from. Both the entry menu and the sidebar show the different functionalities/modules of the application (corresponding to the functionalities specified in 4.1.1.).

The health monitoring module, shown in Figure 3B, represents the screen where the elderly person checks how their health is, whether they need to call their caregiver, or whether everything is fine. They can also check their vital signs, data measured by the wearable. One point made by the caregivers during the user research phase was that the colour red should not be used in this module so that the elderly would not be frightened at any time, as this could have consequences for their health.

**Figure 4:**
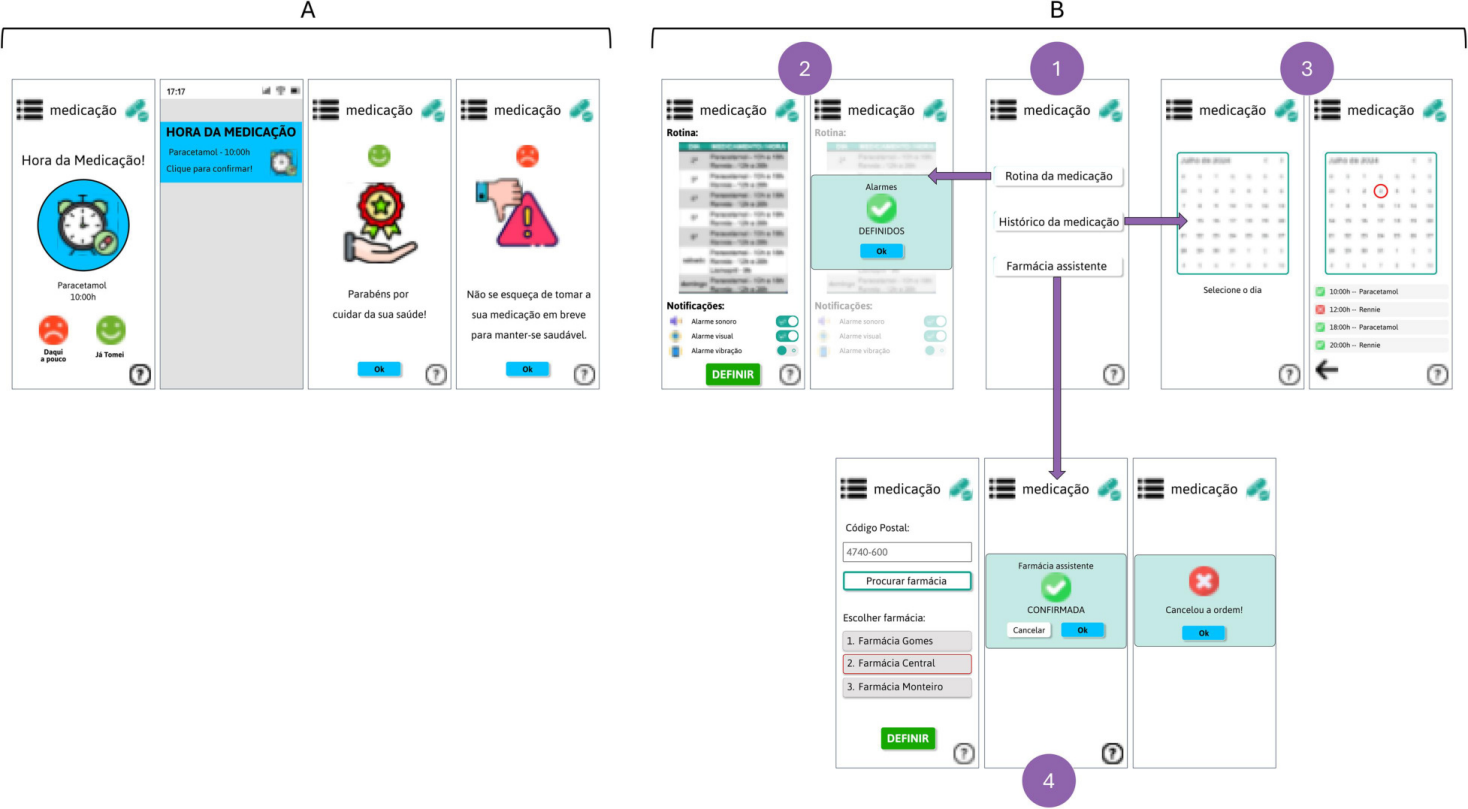
**(A)** Medication intake notification module received by the elderly; **(B)** Medication management module. (Interfaces in Portuguese).

Figure 4A shows the notification received by the elderly person when it is time to take their medication. After opening the notification, they must confirm whether or not they have taken their medication.

Figure 4B shows the medication management module (screen 1), where the elderly person can: consult their medication routine, and also personalise the alarms they receive, audible, visual and vibration (screens 2); check their medication history by day (screens 3); choose an assistant pharmacy (screens 4).

**Figure 5:**
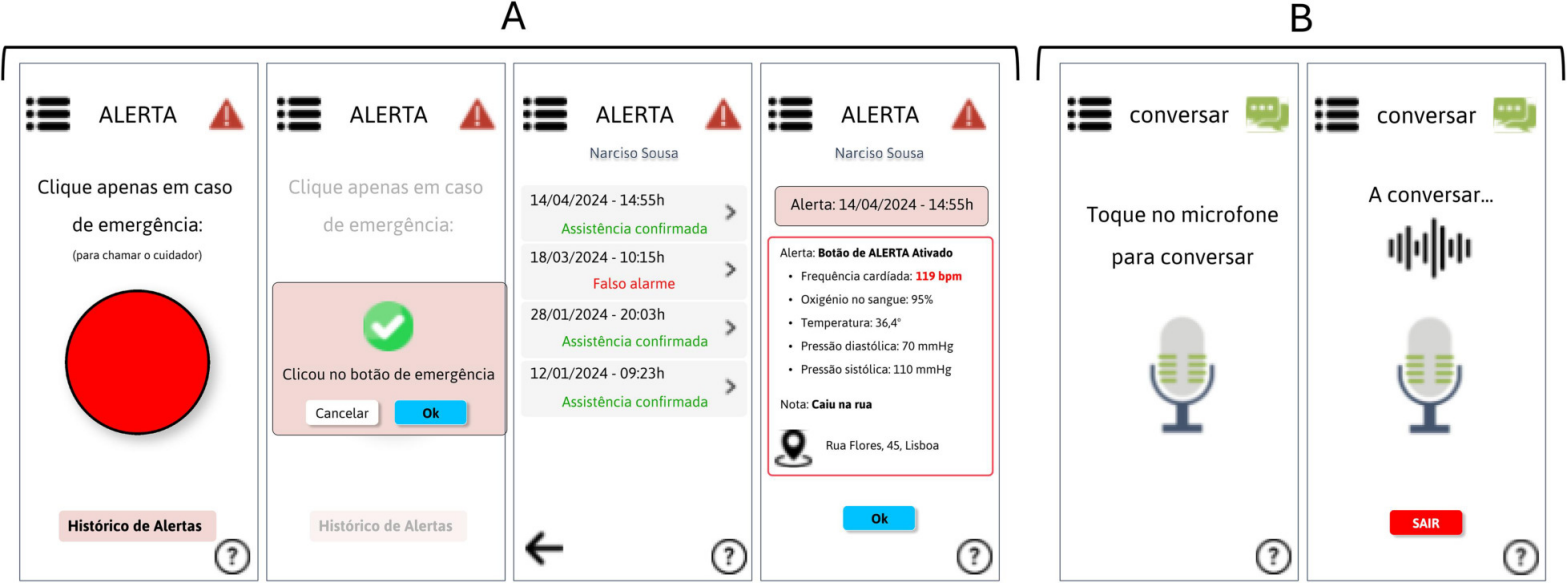
**(A)** Panic/Alert button module; **(B)** Virtual assistance module. (Interfaces in Portuguese).

Figure 5A shows the alert button module, where the elderly person can click in the event of an emergency and can also consult the history of alerts and check whether the caregiver has provided assistance.

The virtual assistance module shown in Figure 5B shows the part of the app where the elderly person can talk to “someone” when they feel the need.

Figure 2 illustrates a conceptual prototype of the wearable device, designed according to the needs identified by the elderly during the user research phase. This model has not yet been materialised in the real world.

##### 4.1.2.2 Caregiver

**Figure 6:**
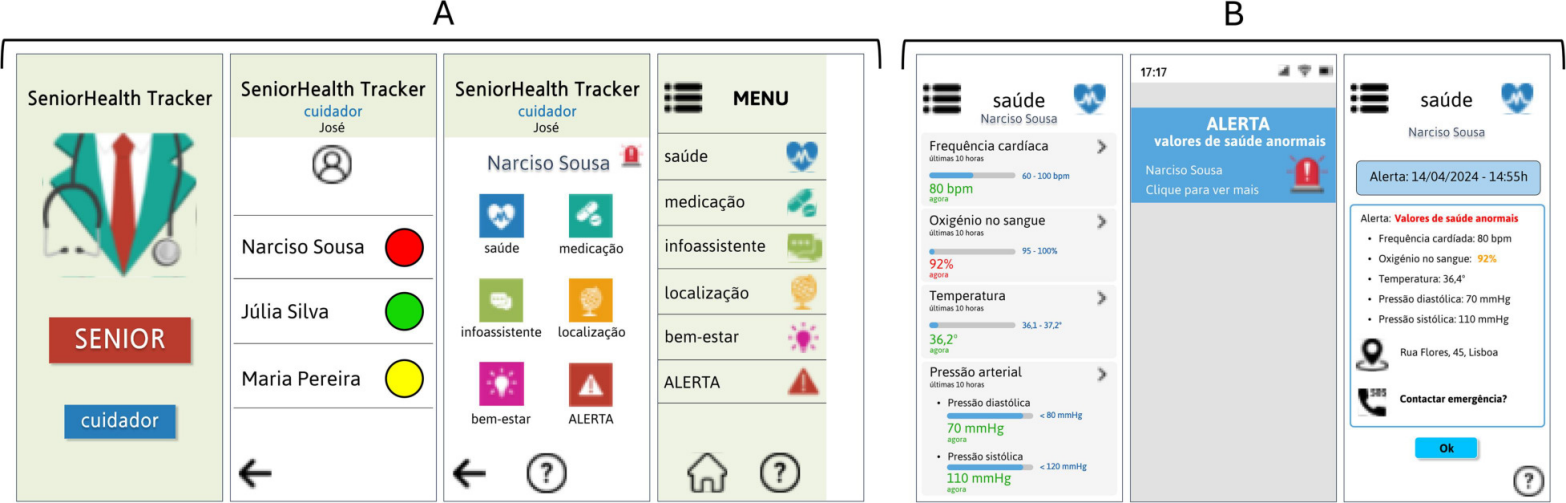
**(A)** Caregiver initial menu and lateral bar; **(B)** Health monitoring module. (Interfaces in Portuguese).

Figure 6A shows the initial screen, the main entrance to the application, where the caregiver chooses “caregiver” and then goes to the menu with the different elderly people they are responsible for. Each one has a red, yellow or green light, depending on how much attention it needs at a specific moment. Both the entry menu and the sidebar show the different functionalities/modules of the application (presented in Section 4.1.1.).

Figure 6B shows the health monitoring module, where the caregiver can check the elderly person's vital signs in real time. In addition, as soon as the values are abnormal, the caregiver receives a notification to keep them alert. They can then check what triggered the alert, see the elderly person's location and contact the emergency services if necessary.

**Figure 7:**
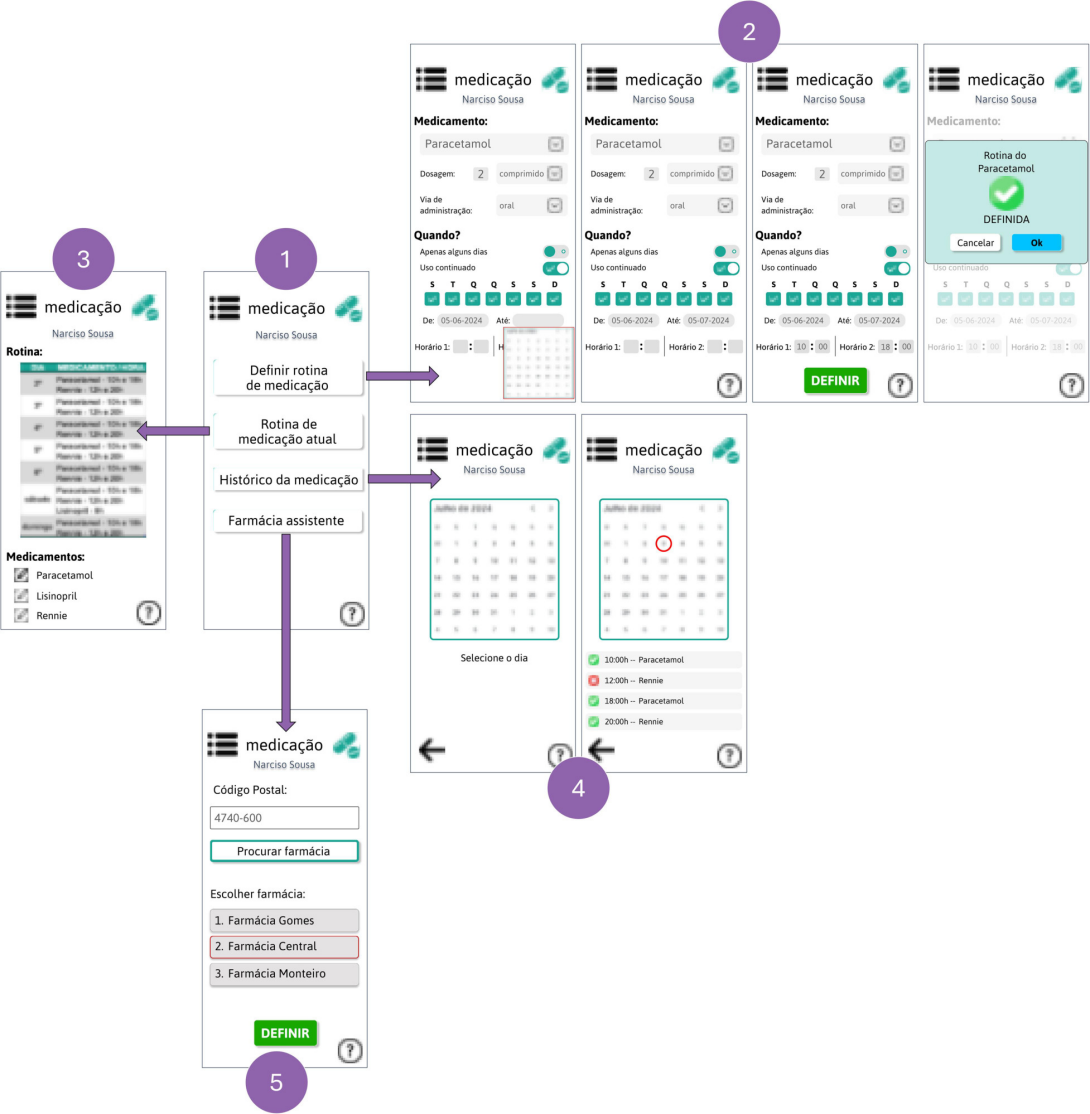
Medication management module. (Interfaces in Portuguese).

Figure 7 shows the medication management module. Screen 1 is the entry menu, where you can choose what you want to do:

define the medication routine, where the app receives the prescription directly from the hospital system and the caregiver simply defines the routine: which drug, the dosage, the route of administration, whether it is to be taken continuously or not, between which days it should be taken, and what time it should be taken (screen 2);
check the current medication routine, with the possibility of editing it (screen 3);
checking the elderly person's adherence to medication, and confirming that they have taken their medication correctly, through the “medication history” (screens 2);
define the elderly person's pharmacy assistant (screen 4).

**Figure 8:**
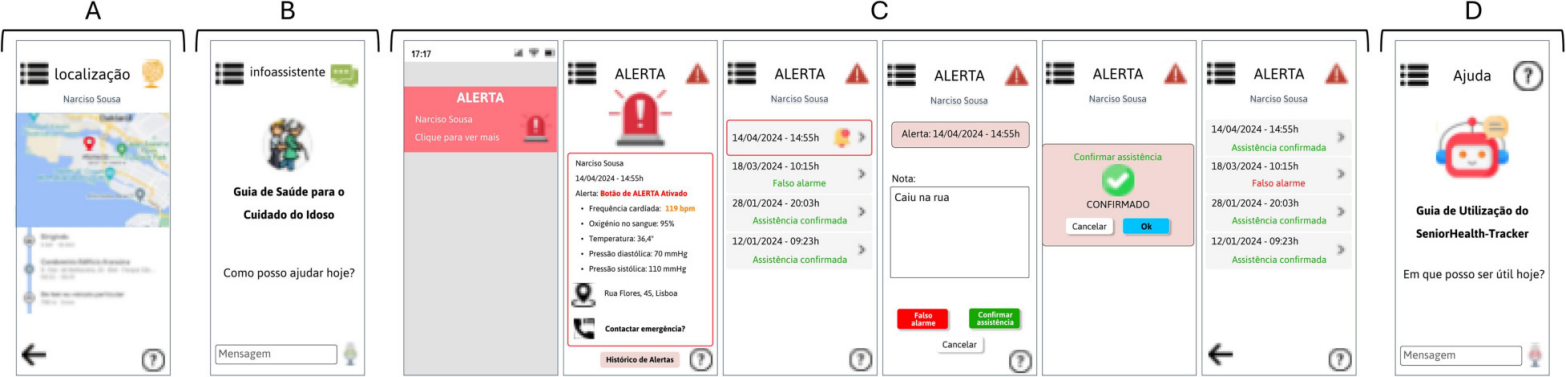
**(A)** GPS location module for the elderly; **(B)** Virtual assistant module; **(C)** Module of the alert button triggered by the elderly person; **(D)** Help module. (Interfaces in Portuguese).

Figure 8A shows the module where the caregiver can check the elderly person's location in real time, with the possibility of consulting the location history.

Figure 8B shows the screen of the virtual assistant where the caregiver can ask questions related to elderly care procedures. This chatbot is based solely on healthcare guidelines for the elderly, avoiding the possibility of passing on unreliable information.

The alert button module, which is triggered by the elderly person, Figure 8C, shows how the caregiver receives a notification on their mobile phone when the elderly person needs assistance and presses the button. This way, the caregiver can quickly check their vital signs, location, and even call the emergency services. In the end, the caregiver must record in the app what triggered this action on the part of the elderly person, and confirm the assistance or record that it was a false alarm, while also being able to check the history of alerts.

Finally, the help module, shown in Figure 8D, represents the screen where the caregiver and the elderly person (screen common to both) can ask any questions about how to use the app via a chatbot.

Following the final iteration, a formative usability evaluation was conducted with older adults and caregivers. Overall, participants tended to rate most tasks as easy to complete, and their feedback informed minor interface refinements. These findings provide initial usability signals for this prototype, but they should be interpreted cautiously given the small, convenience-based sample and the proof-of-concept nature of the study. The results indicate that the requirements identified in the requirements-gathering phase were addressed in the prototype, while further validation is needed in larger and more diverse contexts.

In addition, during the tests, users provided five valuable suggestions for improving the layout of the application and the functionalities themselves, which were implemented, as reflected in the screenshots presented above. The comments obtained through post-test questionnaires indicated general satisfaction with the tool and confirmed its usefulness in supporting the elderly.

### 4.2 mHealth—SeniorHealth Tracker—potential contribution pathways to SDH

The importance of the SDH in mHealths cannot be underestimated, as these determinants significantly influence the health and well-being of the population. Incorporating a thorough understanding of SDH into the development and implementation of mHealth solutions is essential to ensure that these technologies not only promote autonomy and improve access to healthcare but also mitigate existing disparities. By considering these factors, it is possible to create more equitable and inclusive interventions, in line with the principles of a human-centred society (19, 20, 22).

The SeniorHealth Tracker app prototype was developed with a human-centred approach, motivated by the SDH to address the needs of the elderly population. Thus, based on the prototype developed, it has the preliminary potential to support SDH related needs by making it possible to:

**Access to services (potential use-case):** SeniorHealth Tracker technology is intended to facilitate access to medication delivery services, transport to local services and home care, which may support the independence and quality of life of older people, especially those living far from urban centres, in areas considered remote. This topic is in line with the literature that highlights mHealth as a powerful tool for improving access to healthcare in remote or under-served areas, reducing geographical barriers and promoting equity in access (19, 20, 54, 67, 68).
**Access to information (Empowerment) and digital health literacy (intended benefit):** The SeniorHealth Tracker app is intended to provide older people with quick and easy access to information that promotes a healthy lifestyle and knowledge of what to do if their vital signs are abnormal. The wearable device is conceptually designed to help manage and control vital signs, providing potentially useful data for disease prevention and management. This empowerment of patients is consistent with what the literature points to as an advantage of mHealth solutions, encouraging self-management of health and providing understandable and accessible information (67, 68).
**Facilitated communication with health professionals and caregivers (potential pathway):** SeniorHealth Tracker may enable remote communication and follow-up by caregivers, enabling health monitoring and treatment/medication management, which is especially valuable for residents of remote areas. The literature corroborates that mHealth can improve public health surveillance and evidence-based decision-making, contributing to disease prevention and health promotion (54).
**Reducing isolation and social support (aspirational outcome):** SeniorHealth Tracker, with its virtual assistant, is intended to help reduce social isolation by connecting older people with “someone” who responds to them, be it a robot or a support group, promoting social interaction. The literature also emphasises the importance of the social support provided by mHealth technologies (20, 54).
**Emergency Response (intended functionality):** The alert button is intended to improve the safety of the elderly by ensuring that help and medical care may be sought made available in cases of emergency. This response capacity is fundamental to dealing with emergencies efficiently, as indicated by the literature (20).

By addressing SDH, this type of digital solution may contribute to improving access and support for elderly people. Nevertheless, the extent of any impact on health and equity requires empirical evaluation in future, larger-scale studies.

The interviews revealed that not all older people are able to use 100% of the app's functionalities, which could end up catalysing the “digital divide” highlighted by Paglialonga et al. (6). Hendl & Shukla (68) emphasise that the implementation of technology must be accompanied by policies that promote equity in access, something that must be considered in the development and expansion of SeniorHealth Tracker. In summary, the integration of SDH into SeniorHealth Tracker is presented here as a design rationale and longer-term aspiration for a more holistic and equitable use of mHealth technologies for older adults, rather than as a demonstrated outcome of the present study.

## 5 Discussion

This study should be interpreted as an early-stage prototype development and formative UCD effort, focused on feasibility-oriented design choices, usability signals, and early design insights. The SeniorHealth Tracker app is intended to address the technology gap highlighted by the study by Bhayana et al. (29), which showed a significant disparity in the use of technology between the older and younger generations. With the aim of improving the well-being of the elderly, this application offers a set of prototype functionalities., specifically designed to meet the needs and capabilities of this age group, identified through a survey of users and tests carried out by future users. By aiming to support digital inclusion and autonomy, the prototype focuses on feasible, usability-oriented design choices rather than demonstrated health-system outcomes.

In fact, as mentioned in the literature, the continuous advance of medicine and technology has proved to be a catalyst for the ageing of the population, a global trend that is reflected in the annual increase in the number of elderly citizens. However, with this increase in the number of elderly people also comes an increase in the burden on caregivers (15, 55).

SeniorHealth Tracker's functionalities may help to reduce the burden on caregivers, facilitating communication between the caregiver and the elderly person and promoting the elderly person's autonomy. This includes the administration of medication, the monitoring of health parameters, the use of an emergency button and the real-time localisation of the elderly. This study adds value to the literature by presenting a healthcare application that promotes the autonomy of the elderly and improves communication with caregivers, meeting the need for digital solutions emphasised by Kim et al. (17). Additionally, our findings align with literature suggesting that digital tools may support care processes (25–28).

In particular, the functionality of the virtual assistant for the elderly aims to reduce social isolation through digital tools, corroborating the ideas of Kuerbis et al. (24). During the interviews with the elderly, it was possible to understand that from their point of view, a virtual assistant is very useful for offering them company, and they even suggested friends they would definitely like. In the light of the study by Wilson et al. (53), some of the barriers and facilitators identified for the use of technology by the elderly are low self-efficacy, fear of technology and concerns regarding the privacy, confidentiality and security of personal health information. Many older people feel insecure about using new technologies, a barrier that may be mitigated by the familiarity and support provided by a virtual assistant. In addition, the lack of ongoing support is another significant barrier; in this context, the virtual assistant may act as a facilitator, providing ongoing support and helping older people to feel more confident and comfortable using technology.

In the context of ageing, accessibility is inseparable from a humanised approach to digital health. Beyond motivational and emotional barriers, older adults may face visual, cognitive, motor, and digital literacy limitations that directly affect interaction with mHealth solutions. The prototype therefore prioritised low cognitive load (short task flows and consistent navigation), visual clarity (high contrast and simple icon + text cues), and multimodal support (voice and configurable alerts). These decisions aim to reduce technology anxiety, support autonomy, and facilitate safe self-management, while recognising that accessibility needs can vary widely across older adults.

Regarding the privacy, confidentiality and security of personal health information, during the interviews the elderly were asked about their willingness to share this data with their caregivers. Although they answered yes, they were concerned about who else might have access to their personal information. As such, addressing these concerns through robust data protection measures and clear communication about how personal information is handled is intended to reduce perceived barriers and may increase older adults' willingness to engage with technology-facilitated healthcare solutions.

In addition to all this, it was possible to map potential pathways through which the prototype's functionalities could relate to SDH (e.g., access, communication, support), grounded in the literature; these pathways remain hypothetical and should be tested in future real-world evaluations, corroborating the idea put forward by Kumar et al. (20), who argue that technology has great potential to positively influence these determinants. Overall, these results support the relevance of exploring digital solutions for SDH-related challenges, while highlighting the need for larger and more diverse studies to assess real-world impact.

## 6 Final remarks

### 6.1 Conclusions

The article discusses the critical intersection between technological innovation and the human touch, emphasising that while technology provides powerful tools to improve the quality of life of the elderly, it is the human element that transforms these tools into effective solutions. It is important to emphasise that an app for monitoring the elderly must be more than a set of digital functionalities, although these are essential; it must be a gateway to a caring and responsive human service.

The article presents the essential features of an elderly health monitoring app, such as real-time health monitoring, medication management and virtual assistance, the panic/alert button with GPS (allows quick responses in the event of an emergency), mapping of local services, and health and wellbeing tips for the elderly. These features emerged from user research carried out with both caregivers and the elderly themselves, and the app was tested with users, and in this small evaluation participants generally perceived the prototype as useful and easy to use. Given the small, convenience-based sample and the prototype nature of the solution, these findings are preliminary and should not be generalised. Once again, it is necessary to emphasise the value of the human accompaniment behind the technology: health professionals who analyse the data collected, respond to alerts and offer personalised support.

In addition, this technology (app + wearable) can be adapted so that it can be used by people with specialised care needs, further extending its reach and potential for a positive impact on the health and well-being of a variety of populations.

### 6.2 Contributions and implications

#### 6.2.1 Practical implications

The study presents an app prototype for monitoring the health of the elderly, designed to be accessible and easy to use, in order to meet the specific needs of this population. It may also act as an integrating hub for various services: medication management; easier access to health services; improved communication with the caregiver, for early decision-making; remote health monitoring; health literacy/education; and a 24 h virtual assistant, which helps to combat social isolation. This work suggests that combining technology with human support may offer a promising direction for elderly care; however, the present findings are preliminary and future studies are required to evaluate the impact in real-world settings.

By integrating different functionalities into a single app, this study not only highlights the potential of digital technologies to improve healthcare provision for the elderly but also emphasises the importance of inclusive technological approaches adapted to the unique needs and challenges of an increasingly ageing population. Integrating a range of functionalities into a single app is particularly advantageous for older people and their caregivers, as it avoids the need to learn how to use several different platforms, thus reducing the learning curve and facilitating ongoing use of the same tool.

#### 6.2.2 Theoretical implications

This study contributes to the existing literature by drawing on the SDH framework to outline potential pathways through which health apps, such as the SeniorHealth Tracker, may support access to healthcare and, in the longer term, help inform efforts to reduce access disparities among older adults.

Thus, the study not only informs on suitable technological design considerations for older adults but also outlines how future technological interventions could be strategically implemented to address SDH related barriers in ageing populations, subject to empirical evaluation.

### 6.3 Limitations and future work

#### 6.3.1 Limitations of the study

A key limitation of this study is the small, convenience-based and geographically limited sample used in the requirements-gathering stage (4 interviews with older adults and 4 interviews with caregivers), which restricts representativeness and generalisability. While these interviews provided valuable initial insights, the sample may not fully reflect the diversity of experiences, needs and expectations across the wider older adult population. As this was an early exploratory User-Centred Design phase, the aim was to elicit initial needs and usability considerations to inform personas, scenarios and a first prototype iteration, rather than to support population-level conclusions.

Although accessibility was considered in the prototype design, a formal accessibility evaluation (e.g., systematic assessment of readability, touch-target sizing, and performance with users with visual/cognitive/motor impairments or low digital literacy) was not conducted in this preliminary proof-of-concept phase.

In addition, it is important to mention that not all older people have access to technological devices or high-speed internet, especially in rural areas or for those with limited financial resources, which could create a disparity in access to the benefits of the app.

What's more, cultural and linguistic diversity among the elderly can affect how they perceive and use technology and can also limit the effectiveness of the app in different cultural contexts.

#### 6.3.2 Future work

As future work, and to deepen and broaden the results of this study, it is essential to:

***Expand interviews***: Carry out interviews with a significantly larger number of participants, covering both older people and healthcare providers. These interviews should be conducted in different geographical regions and socio-economic contexts, to obtain a more comprehensive and representative view of needs and perceptions in relation to technology.
***Accessibility evaluation:*** Future work should include dedicated accessibility testing with a larger and more diverse sample (including users with visual, cognitive, motor, and low digital literacy limitations) and, where possible, alignment with recognised accessibility guidelines.
***Develop interdisciplinary partnerships***: In addition, the authors plan to continue developing partnerships with specialists in gerontology, information technology and user experience design. These interdisciplinary collaborations aim to create a holistic approach to improving and implementing the technology, ensuring that it more effectively and efficiently meets the needs of the target audience.
***Establish a continuous feedback mechanism***: Also, it is essential to establish an ongoing feedback mechanism with users. This mechanism will allow the application to be updated based on the suggestions and needs of the elderly and healthcare providers.
***Feasibility and constraints of the wearable concept.*** The wearable component remains conceptual and has not yet been engineered or validated. Practical constraints include sensor selection and calibration, battery autonomy, ergonomics and comfort, safe medication compartment design, durability, connectivity reliability, and data security. Future work will require iterative hardware prototyping, safety/risk assessment, and validation in real-world contexts before clinical or operational deployment.
***Integrate of functionalities suggested by health professionals***: Finally, the plan is to integrate features suggested by a group of healthcare professionals, including the possibility of personalising health monitoring with specific fields for vital signs. This innovation will allow users, especially elderly people with chronic conditions such as diabetes, coagulation problems, among others, to autonomously record their observations throughout the day. The flexibility of this self-monitoring feature is crucial, since the elderly population often has multiple comorbidities, thus increasing the reach and relevance of the application to a wider target audience.

Another suggestion given by health professionals would be for SOS medication, i.e., above a certain health value (i.e., blood pressure, temperature,…), the elderly person would receive an alert notification to take SOS medication to control the value.

## Data Availability

The original contributions presented in the study are included in the article, further inquiries can be directed to the corresponding author/s.
